# Conversion of M1 Macrophages to Foam Cells: Transcriptome Differences Determined by Sex

**DOI:** 10.3390/biomedicines11020490

**Published:** 2023-02-08

**Authors:** Rafael Nambo-Venegas, Berenice Palacios-González, Jaime Mas-Oliva, Ana Karen Aurioles-Amozurrutia, Armando Cruz-Rangel, Abel Moreno, Alfredo Hidalgo-Miranda, Mauricio Rodríguez-Dorantes, Felipe Vadillo-Ortega, Juan Xicohtencatl-Cortes, María Isabel Ruiz-Olmedo, Juan Pablo Reyes-Grajeda

**Affiliations:** 1 Laboratorio de Estructura de Proteínas, Instituto Nacional de Medicina Genómica, Mexico City 14600, Mexico; 2 Laboratorio de Envejecimiento Saludable, Centro de Investigación Sobre Envejecimiento (CIE-CINVESTAV Sur), Instituto Nacional de Medicina Genómica, Mexico City 14330, Mexico; 3 Instituto de Fisiología Celular, Universidad Nacional Autónoma de México, Mexico City 04510, Mexico; 4 Instituto de Química, Universidad Nacional Autónoma de México, Mexico City 04510, Mexico; 5 Laboratorio de Genómica del Cáncer, Instituto Nacional de Medicina Genómica, Mexico City 14600, Mexico; 6 Laboratorio de Oncogenomica, Instituto Nacional de Medicina Genómica, Mexico City 14600, Mexico; 7 Unidad de Vinculación Científica de la Facultad de Medicina UNAM en INMEGEN, Instituto Nacional de Medicina Genómica, Mexico City 14600, Mexico; 8 Laboratorio de Bacteriología Intestinal, Hospital Infantil de México Federico Gómez, Mexico City 06720, Mexico; 9 ROPPEN R&T de R.L. de C.V., Mexico City 14650, Mexico

**Keywords:** LDL internalization, M1 macrophages, sex-related, transcriptional profile, chromosome Y, female, atherosclerosis

## Abstract

Background: M1 macrophages involved in pro-inflammatory processes can be induced by low-density lipoproteins (LDL), giving rise to foam cells. In the atheroma plaque, it has been identified that males present more advanced lesions associated with infiltration. Therefore, our study aims to investigate sex-related changes in the transcriptome of M1 macrophages during the internalization process of LDL particles. Methods: Peripheral blood mononuclear cells (PBMCs) from healthy male and female subjects were separated using Hystopaque, and monocytes were isolated from PBMCs using a positive selection of CD14+ cells. Cells were stimulated with LDL 10 µg/mL, and the transcriptional profile of M1 macrophages performed during LDL internalization was determined using a Clariom D platform array. Results: Chromosome Y influences the immune system and inflammatory responses in males expressing 43% of transcripts in response to LDL treatment. Males and females share 15 transcripts, where most correspond to non-coding elements involved in oxidative stress and endothelial damage. Conclusions: During LDL internalization, male monocyte-derived M1 macrophages display more marked proinflammatory gene expression. In contrast, female M1 macrophages display a more significant number of markers associated with cell damage.

## 1. Introduction

Cardiovascular disease (CVD) still represents the leading cause of death in Mexico [1], where atherosclerosis, considered a progressive condition characterized by the accumulation of lipids and fibrous components preferentially in the large arteries, represents the leading cause of CVD. The progressive chronic inflammatory process in the lamina of the arteria results in atheroma plaque, initiated by the subendothelial retention of low-density lipoproteins (LDL). Otherwise, the accumulation of macrophages (MACs) in the endothelium induces an inflammatory response [2], which promotes the progression of the atherosclerotic process. In addition, the LDL uptake by endothelial cells triggers the production of several pro-inflammatory molecules, including macrophage colony-stimulating factor (M-CSF) and adhesion molecules [3,4].

Interestingly, although it has been recognized for some time now that the presence of autoimmune disease in women increases the risk of coronary artery disease compared to men, women are less likely to develop atherosclerosis [5]. Atherosclerosis in women has been associated with autoimmune diseases such as rheumatoid arthritis, systemic lupus erythematosus, and systemic sclerosis. These findings have promoted the concept that men’s immune mechanisms that lead to coronary artery disease are different from those in women. For instance, the presence of atheroma lesions in men has been associated with a higher infiltration of M1 macrophages and higher levels of iron stores in the blood, unlike in women [6].

Macrophages belong to a group of pleiotropic cells [7] whose activation is identified by markers such as CD163, sCD14, Gal3BP, sCD25, and sCD166 [8]. In the atherosclerotic plaque, two groups of macrophages coexist, mainly M1 and M2. M1 macrophages are known to be activated by lipopolysaccharides (LPS) and inflammatory cytokines such as interferon-gamma (IFNγ) and synthesize a series of chemokines to recruit additional leukocytes. Moreover, M1 macrophages produce high inducible nitric oxide synthase (iNOS) and pro-inflammatory cytokines such as IL-1, IL-6, IL-12, and tumor necrosis factor-alpha (TNF-α). Activation by IL-4 and IL-3 generates the polarization of monocyte/macrophages to be converted to M2 macrophages, which promote anti-inflammatory responses characterized by an increase in arginase 1 (Arg1) and the expression of the CD206 mannose receptor [7]. 

Several studies investigating atherosclerotic plaque recognized a high content of M1 macrophages and lower content of M2 macrophages, located far from the lipid core and containing fewer lipids [9], where the phenotype control resides on the lesion microenvironment. Moreover, unlike M2 macrophages, M1 macrophages are present in symptomatic patients with unstable plaque [10]. In atherosclerosis, it has been well-defined that chemically modified lipoproteins associated with oxidative stress trigger an innate immune response carried out by macrophages that subsequently undergo an adaptive immune response influenced by the presence of cholesterol, phenotypic plasticity, metabolism, age, and sex [11].

Since, nowadays, although the literature presents scattered data, there is still a lack of information that might integrate the M1 macrophage into the development of gender-related atherosclerosis, the present study analyzes differences found during LDL internalization according to sex.

## 2. Materials and Methods

### 2.1. Study Design

This study was designed to investigate sex-related variations in the transcriptome of M1 macrophages during the LDL internalization process. Healthy plasma donors were recruited in an anonymized form at the Institute’s central blood bank. The study was approved by the National Institute of Genomic Medicine (25/2011/I).

### 2.2. Low-Density Lipoprotein (LDL) Isolation

LDL was isolated from the fasting plasma of normolipidemic volunteers. LDL was separated by discontinuous density gradient ultracentrifugation at 657,000× *g* for 2.5 h at 10 °C. The supernatant was diluted 1:4 in PBS (Gibco, 70011, Waltham, MA, USA) and centrifuged at 657,000× *g* for 2.5 h at 10 °C. Lipid fraction was resuspended in 2 mL of PBS. Finally, LDL was washed by ultracentrifugation at 657,000× *g* for 2.5 h at 10 °C and resuspended in 1 mL of PBS (Gibco, 70011, MA, USA). LDL was quantified using the 2D-Quant KIT (GE, 80648356, Arlington Heights, IL, USA) according to the manufacturer’s instructions. The LDL fraction was confirmed by HPLC (Supplementary Materials; Figure S1). Briefly, a 1:10 dilution of the LDL/PBS sample was performed, and the dilution was filtered (0.2 μm, Nalgene,171-0020, Rochester, NY, USA). An HPLC (Waters, 2695-2996-2475, Milford, MA, USA) and a Bio-Sil Gel Filtration column (Bio-Rad, 125-0062, Heracles, CA, USA) were used with an isocratic PBS gradient at a flow rate of 1 mL/min, 1000 psi, 25 °C, detected at 220 or 280 nm while scanning within 110–350 nm. Validation was carried out employing an LDL/DiI complex (Invitrogen, L3482, Waltham, MA, USA) employed using the exact same conditions (excitation 554 nm and emission 571 nm). LDL was quantified using the 2D-Quant KIT (GE, 80648356, IL, USA) according to the manufacturer’s instructions.

### 2.3. In Vitro Isolation and Cultivation of Monocytes

Peripheral blood mononuclear cells (PBMC) were separated by centrifugation at 1,222,533× *g* for 10 min in a Sorvail Legend RT Centrifuge (Thermo Scientific, 75004367, Waltham, MA USA) when the three phases were formed: aqueous (plasma), interface (buffy coat), and precipitate (erythrocytes). The buffy coat was collected in a new tube to which 10 mL of PBS (Gibco, 70011, MA, USA) was added. Previously, 3 mL of Hystopaque (Sigma, 10771, Medford, MA, USA) was placed in another new tube and the mixture was added taking care not to break the phases; it was centrifuged without brake at 1509.3× *g* for 30 min at 10 °C. The PBMC fraction was collected and subsequently placed in a new tube, 10 mL of PBS was added and centrifuged at 167.7× *g* for 1 min, then the supernatant was decanted, and the washing was repeated 3 times.

The monocytes were isolated from PBMCs using EasySep™ Human CD14 Positive Selection Kit II (STEMCELL TECHNOLOGIES, 17858, YVR, Vancouver, BC, Canada), obtaining purity greater than 97%. After isolation, cells were cultured at 37 °C in RPMI + L-Glutamine (Gibco, 21127, MA, USA) supplemented with 10% Fetal Bovine Serum (ATCC, 30-2020, Manassas, VA, USA) and 1% antibiotic (Pen Strp. Gibco 15140, MA, USA) in 6-well plates (CellBind Surface, Corning, 3335, Somerville, MA, USA). After 4 h, non-adherent cells were removed, and RPMI + L-Glutamine (Gibco, 21127, MA, USA) supplemented with 10% Fetal Bovine Serum (ATCC, 30-2020, VA, USA), 1% antibiotic (Pen Strp. Gibco 15140, MA, USA), and 5 ng/mL M-CSF (Sigma, M6518, MA, USA) was added and maintained for seven days. Subsequently, the macrophages were polarized to subpopulation M1 by adding interferon-gamma (INFɣ) 20 ng/mL (Millipore, IF002, Burlington, MA, USA) and lipopolysaccharide 100 ng/mL (SIGMA, L4391-1MG, MA, USA).

### 2.4. Macrophage Differentiation and LDL Treatment

To M1 macrophage polarization, monocytes culture medium was removed and replaced with RPMI + L-Glutamine medium (Gibco, 21127, MA, USA) with 10% Fetal Bovine Serum (ATCC, 30-2020, VA, USA), 1% antibiotic (Pen Strp. Gibco15140, MA, USA), supplementing with interferon-gamma (INFɣ) 20 ng/mL (Millipore, IF002, MA, USA) lipopolysaccharide 100 ng/mL (Sigma, L4391-1 MG, MA, USA) maintaining the standard conditions of 36.5 °C and 5% CO_2_ for 24 h.

M1 macrophages were further stimulated with a single dose of LDL (10 µg/mL) and the transcriptome was evaluated at different times (0, 24, 48, and 72 h) in each study group. RPMI 1640 supplemented with 5% FBS, and IFN-γ (20 ng/mL) lipopolysaccharide 100 ng/mL (Sigma, L4391-1MG, MA, USA) maintaining the standard conditions of 36.5 °C and 5% CO_2_. The confluence in each technical replicate was 200,000 cells per well and they were performed in triplicate for each volunteer. Cell counts were performed using the TC20™ automated cell counter (Bio-Rad, 145-0102, CA, USA).

### 2.5. Microarray Expression and Analysis

Total RNA was isolated with TRIzol™ (Invitrogen, 15596018, MA, USA) according to the manufacturer’s instructions. RNA was quantified spectrophotometrically with NanoDrop (Thermo Fisher Scientific, ND2000CLAPTOP, MA, USA). The quality of RNA was assessed with an Agilent 2100 Bioanalyzer (Agilent Technologies, G2939BA, Santa Clara, CA, USA). Isolated total RNA was amplified, labeled, and hybridized using the Clariom D Clariom™ D Human Array de Affymetrix, (Thermo Fisher Scientific, 902923, MA, USA) following the manufacturer’s instructions. Raw data were analyzed using Affymetrix Expression Console and Transcriptome Analysis Console Software. RNAs with ≥ 2-fold-change (FC), *p* < 0.05, and FDR < 0.7 were selected as being significantly differentially expressed. For the technical validation of the microarray, a Q-PCR analysis was performed for the CD36, FAB, and IL1β genes. (Supplementary Figure S2).

### 2.6. Ingenuity Pathway Analysis (IPA)

Ingenuity Pathway Analysis software (IPA, QIAGEN, Hilden, Germany) was used to identify enriched pathways between males and females (cutoff values: FDR < 0.01; FC > 2, *p* < 0.05), canonical pathways with enrichment score and *p* < 0.05 with greater than 10 gene members and to identify differentially enriched pathways among treatment-time (cutoff values: FDR < 0.05; fold-change >2).

### 2.7. Statistical Analyses

FDR was computed using the Benjamin–Hochberg algorithm. All other data are presented as means ± SEM. For comparison of multiple conditions, data were analyzed by one-way analysis of variance (ANOVA) (95% confidence interval) with Holm–Sidak correction (for multiple comparisons) or Dunnett’s correction (for multiple comparisons to a single control). For comparison of two conditions, data were analyzed by two-tailed unpaired Student’s *t*-test (with Holm–Sidak correction for multiple testing). Statistical analyses were performed using R 4.1.0. Three subjects were used per group in each experiment, and experiments were performed at least three times. To denote significance, * *p* < 0.05, ** *p* < 0.01, *** *p* < 0.001. Graphs were plotted using Database for Annotation, Visualization, and Integrated Discovery (DAVID) online tool version 6.8 and IPA software (IPA, QIAGEN, Hilden, Germany).

## 3. Results

### 3.1. Transcriptomic Profile of M1 Macrophages Stimulated with LDL Associated with Sex

To observe sex-dependent changes in LDL internalization in activated M1 macrophages becoming foam cells, the transcriptome of M1 macrophages treated with 10 µg/mL of LDL obtained from healthy males was compared under the same conditions to M1 macrophages obtained from healthy females. Differentially expressed genes as twofold values are shown in Table S1. One hundred eighteen genes were found to be differentially expressed between males and females: Multiple Complex 37; Coding 37; Pseudogene 2; Non-coding 28; Precursor microRNA 3; and unassigned 11. Table 1 shows gene expression in chromosome Y. Genes encoding cellular adhesion molecules (NLGN4Y), cellular exocytosis (TXLNGY), associated with the induction of IFN-α (DDX3Y), cellular apoptosis (EF1AY), and prevention of protein degradation (USP9Y) are shown to be expressed in agreement with the pathophysiology of atherosclerosis. 

### 3.2. Pathways in M1 Macrophages Stimulated with LDL Associated with Sex and Time

**Figure 1:**
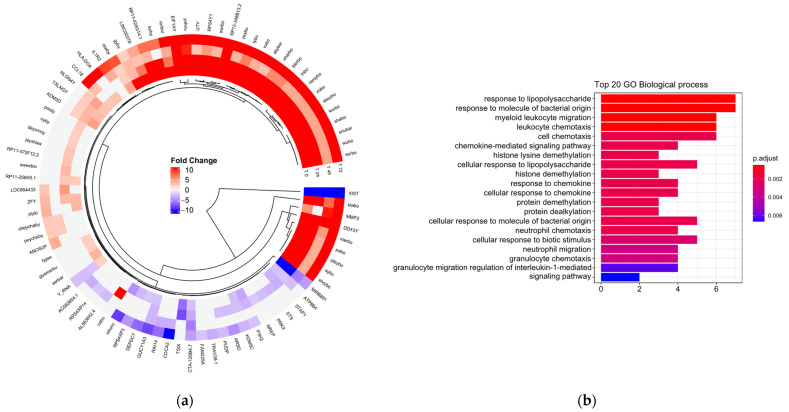
(**a**) Circular heat map representation of unsupervised hierarchical clustering from analysis of different time (rows) grouped by gene (columns). The red tones indicate upregulation and blue tones, downregulation. (**b**) Gene ontology (GO) enrichment between men and women. Top 20 significantly enriched GO. *p*.adjust (adjusted *p*-value): Red < purple < blue. Graphs were plotted using Database for Annotation, Visualization, and Integrated Discovery (DAVID) online tool version 6.8.

Subsequently, it was evaluated whether there were differences in gene expression dependent on exposure to LDL (Figure 1a). The main functions associated with the atherosclerotic process were the activation and chemotaxis of leukocytes involved in cell trafficking and the inflammatory response (Figure 1b). The transcripts involved in these biological processes were CD14, IL6, MICA, CXCL2, EDN1, CCL18, CXCL3, HLA-DOA, IL1R2, SMAD6, and PRKX.

### 3.3. Gene Network between Men and Women in M1 Macrophages Stimulated with LDL

**Figure 2:**
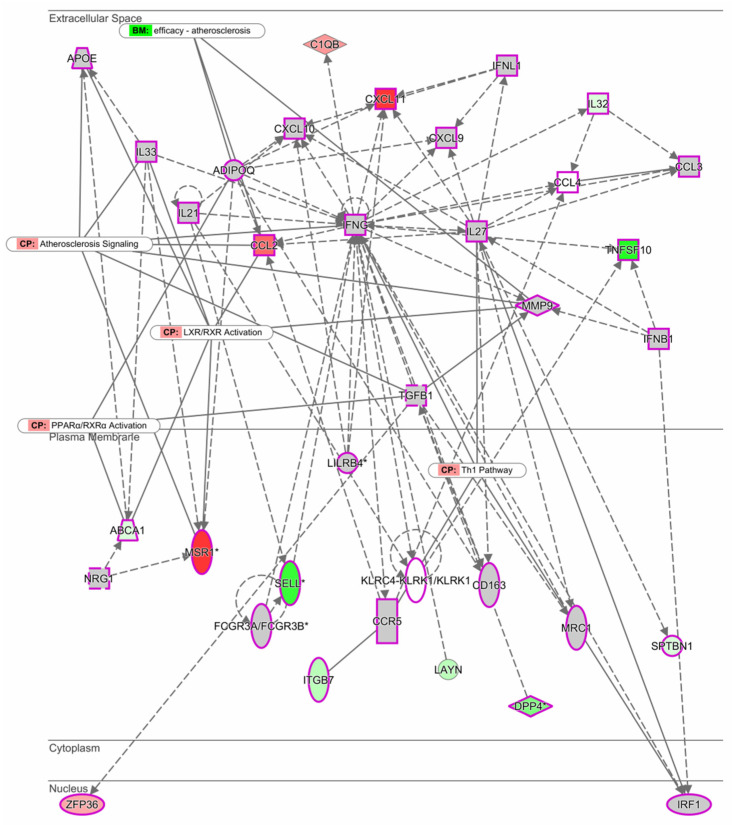
Expression network between men and women. The intensity of the node color-(red) indicated the degree of up-regulation. Genes in uncolored notes were not identified as differentially expressed in our experiment and were integrated into the computationally generated networks based on the evidence stored in the Ingenuity Pathway Analysis (IPA software, QIAGEN). The node shapes denote enzymes (♦), G-protein coupled receptor (▯), transmembrane receptor (0), cytokines (□), growth factor (⬚), transporter (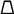), and other (◯). Graphs were plotted using IPA software by QIAGEN.

Using the Ingenuity Pathway Analysis (IPA) software, we investigated the most significant gene network of M1 macrophages stimulated with LDL between males and females. Interestingly, it was found that under these conditions, the network is organized around the INFγ gene (Figure 2). The top functions related to the INFγ gene correspond to molecules involved in the immune response, cellular movement, cell signaling, and molecular transport. Genes MSR1, CCL2, and CXCL known to be involved in the development of atherosclerosis through the activation of LXR/RXR and PPARα were upregulated.

### 3.4. Biological Processes Associated with Cellular Functions

**Figure 3:**
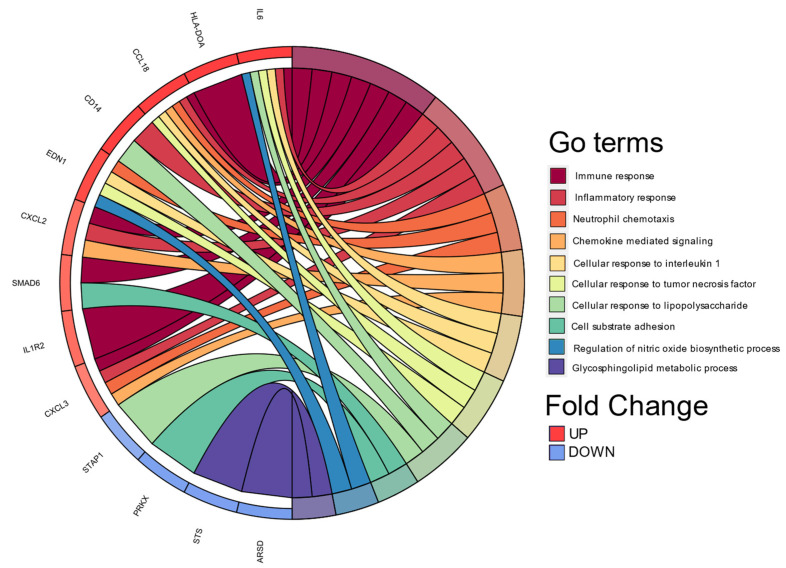
Chord plot of the top 10 Gene Ontology (GO) terms. In each chord diagram, genes contributing to their respective enrichment are shown on the left, and enriched GO clusters are shown on the right. Downregulated mRNAs are displayed in blue, whereas upregulated mRNAs are displayed in red. Each GO term is represented by one colored line. Graph was plotted using Database for Annotation, Visualization, and Integrated Discovery (DAVID) online tool version 6.8.

Continuing with the analyzes to biologically explain the differences in gene expression, we performed a classification based on the co-occurrence of genes to discover biological processes associated with cellular functions and pathways, DAVID 6.8 allowed us to perform it in Gene Ontology Term Enrichment (Figure 3).

## 4. Discussion

Recently, interest has grown in studying sexual dimorphism in the cardiovascular system. Chromosome Y differs from all other human chromosomes in several ways; some examples are that it (1) is the chromosome involved in determining sex (male); (2) does not present recombination; (3) presents a common ancestry, and (4) presents a meiotic relationship with chromosome X [12]. Although an association between gene expression and sex-based differential selection has been previously recognized, it is limited to animal studies due to the lack of large-scale transcriptome sequencing in humans [13,14].

The present study identifies genes already classified in the atherosclerosis process as genes that codify for encoding cellular adhesion molecules (NLGN4Y), cellular exocytosis (TXLNGY), induction of IFN-α (DDX3Y), cellular apoptosis (EF1AY), and prevention of protein degradation (USP9Y) [15]. In addition, an expression profiling study based on new-onset heart failures demonstrated that DDX3Y, EIF1AY, and USP9Y are upregulated in male subjects [16]. 

Evidence indicates that immune and inflammatory responses are influenced by chromosome Y, resulting in programmed susceptibility in men to diseases with immunological components [17]. The present study identifies several genes associated with the immune system, including CXCL3 and CXCL2, linked to immunoregulatory and inflammatory processes; IL-6, which codifies for a cytokine that regulates the inflammation process and maturation of B cells; INHBA, encoding for TGF-β (transforming growth factor-beta), whose function is to regulate the secretion of the follicle-stimulating hormone (FSH) associated with the development of atherosclerosis in postmenopausal women [18]; UTY, whose downregulation in macrophages has been associated with coronary artery disease [19]; DDX3Y, expressed in blood cells that encode for the human male-specific minor histocompatibility antigen [20]; and KDM5D, increased in several atherosclerotic models [21]. 

On the other hand, long non-coding RNAs (lncRNAs) have been implicated in ischemic heart diseases [22]. The present study shows that chromosomal-Y lncRNA TTTY15 and lncRNA XIST (X-inactive specific transcript) are upregulated. Interestingly, while lncRNA TTTY15 preserves cardiomyocytes from hypoxia-induced cell injury, silencing lncRNA TTTY15 inhibits cell apoptosis and preserves cell migration [23]. Regarding XIST, this lncRNA serves as scaffolding for protein recruitment and as a master regulator of X inactivation in mammals. Furthermore, it has been demonstrated that the knockdown of XIST protects endothelial cells from ox-LDL-induced injury and apoptosis [24].

Our canonical pathway and gene network analysis revealed that “chemokine signaling” was an important pathway modulated by upregulated male genes. One crucial gene network was identified around IFN-γ, considered a pro-atherogenic cytokine that promotes the expression of pro-inflammatory cytokines, adhesion molecules, and several chemokines. This gene has also been reported to modulate macrophage differentiation to M1. The present study indicated that around the IFN-γ network, CCL2 and CXCL11 were upregulated, and TNFSF10 (also known as TRAIL), transmembrane receptor SELL, and DPP4 were downregulated. CCL2 is one of the first chemokines identified in atherosclerotic lesions and is mainly produced by monocytes, macrophages, endothelial cells, and smooth muscle cells [25]. CXCL11 can be detected in all stages of plaque development, and evidence suggests that together with chemokines, CXCL9/MIG and CXCL11/ITAC, it regulates T-cell trafficking in atherosclerosis [26,27]. In humans, TRAIL induces apoptosis when associated with the death receptor-4 and -5. TRAIL is mainly expressed in the endothelium, smooth muscle cells, and macrophages within plaques and attenuates atheromatous lesion formation. The underlying mechanism is that TRAIL promotes vascular cell apoptosis in response to a mild dietary fat stimulus [28].

Meanwhile, SELL, also known as CD62L, is a cell surface component that is a member of a family of adhesion/homing receptors that mediates the initial attachment of leukocytes to activated endothelium, representing the first step of leukocyte migration into sites of inflammation [29]. Lastly, DPP4 inhibition may reduce monocyte migration to atherosclerotic plaque in response to TNFα and soluble DPP4. However, inhibition of DPP4 also exacerbates cardiovascular disease by enhancing sympathetic activation and angiogenesis [30]. 

Another of the gene networks identified was around ADIPOQ, known to inhibit CXCR3 ligand production in macrophages. MSR1 and CCL2 were also upregulated around ADIPOQ. MSR1 participates in cell adherence, activation, and foam cell formation, processes involved in atherosclerosis development and progression [31].

When LDL internalization was evaluated, 15 genes were shared along the time course evaluated, and nearly half were non-coding. As mentioned above, EIF1AY, XIST, DDX3Y, KDM5C, and RPS4Y1 have been shown to participate in employing several atherosclerotic models. Septin 4 is a cytoskeleton component implicated in oxidative stress-induced endothelial cell injury. A knockdown model has demonstrated that Septin 4 significantly relieves endothelial apoptosis [32].

Our data add to the evidence that human chromosome Y plays an important role in cardiovascular disease in a sex-specific manner and therefore provides a novel insight into potential new therapeutic targets for atherosclerosis. Some of the limitations of the present study correspond to the fact that it is a descriptive study based on transcripts analyzed limited to the Clariom D microarray, which contains many genes whose function is still unknown.

## 5. Conclusions

During LDL internalization, male monocyte-derived M1 macrophages display more marked proinflammatory gene expression. In contrast, female M1 macrophages display a more significant number of markers associated with cell damage.

## Figures and Tables

**Table 1:** Transcripts expressed in Y chromosome.

Function	Genes	Fold Change
Histone demethylase and inductive activity of INF-α	DDX3Y	254.51
Cellular apoptosis and translation factors	EIF1AY	108.43
Ribosomal protein translation	RPS4Y1	44.91
Unknown	ANOS2P, merbo, RP11-424G14.1, sybo, yohiru, blabo, nabo, tobo, zeybu, blerbo, Serbar, nyby, Shabo, tybo, ZFY-AS1; AC006157.4, pabo, Sharbo, zobo, gyby, pleybo, Shorbo, TC0Y00006490.hg.1, rarsybo, skeybo, vubo, TC0Y00007072.hg.1, korbo, rawby, skybor, warbo, TC0Y00007073.hg.1, rorbor, Snubar, wubo, TC0Y00007286.hg.1, lorby, RP11256K9.1, Sorbo, TC0Y00007293.hg.1, TC0Y00007306.hg.1, Y_RNA.	38.99
Chromatin organization	UTY, KDM5D,	26.3
Transcription regulator	UTY, ZFY	13.26
Long noncoding RNA	TTTY15, LINC00278	6.5
Prevention of protein degradation	USP9Y	4.73
Cellular exocytosis	TXLNGY	2.92
Adhesion molecules	NLGN4Y (Neuroligin 4 Y-Linked)	2.24

One-Way Repeated Measure ANOVA (paired) Fold Change (linear) <2 or Fold Change (linear) >2 ANOVA *p*-value (Condition pair) <0.05.

## Data Availability

The datasets analyzed in this study are available from the corresponding author on reasonable request.

## Supplementary Materials

The following supporting information can be downloaded at https://www.mdpi.com/article/10.3390/biomedicines11020490/s1, Figure S1: Evaluation of the purity of native LDL isolation by high-resolution liquid chromatography. Table S1: Total differential transcripts between men and women. Microarray validation of the, Figure S2: Q-PCR CD36, FAB, and IL1β genes.
