# Supplementary material for: Conversion of M1 Macrophages to Foam Cells: Transcriptome Differences Determined by Sex
Source: Biomedicines. 2023 Feb 8;11(2):490. doi: 10.3390/biomedicines11020490 (PMC9953229; doi:10.3390/biomedicines11020490)
Supplement: Supplementary file 1 [file biomedicines-11-00490-s001.zip › biomedicines-2158916-supplementary.pdf]

*biomedicines*

MDPI

Evaluation of the purity of native LDL isolation by high-resolution liquid chromatography
The retention time of the extract coincides with the commercial LDL-Dil Complex chromatogram, the peak of the signal at 220 and 280nm during the absorbance screening PDA 210 to 400nm at 1.2nm is like that evaluated in reference fluorescence Ex554nm-Em571nm. No protein signal was observed for 30 minutes that are enough for similar protein analyzes with a C18 column and the same conditions 1ml / min isocratic PBS, 1000psi 25ºC.

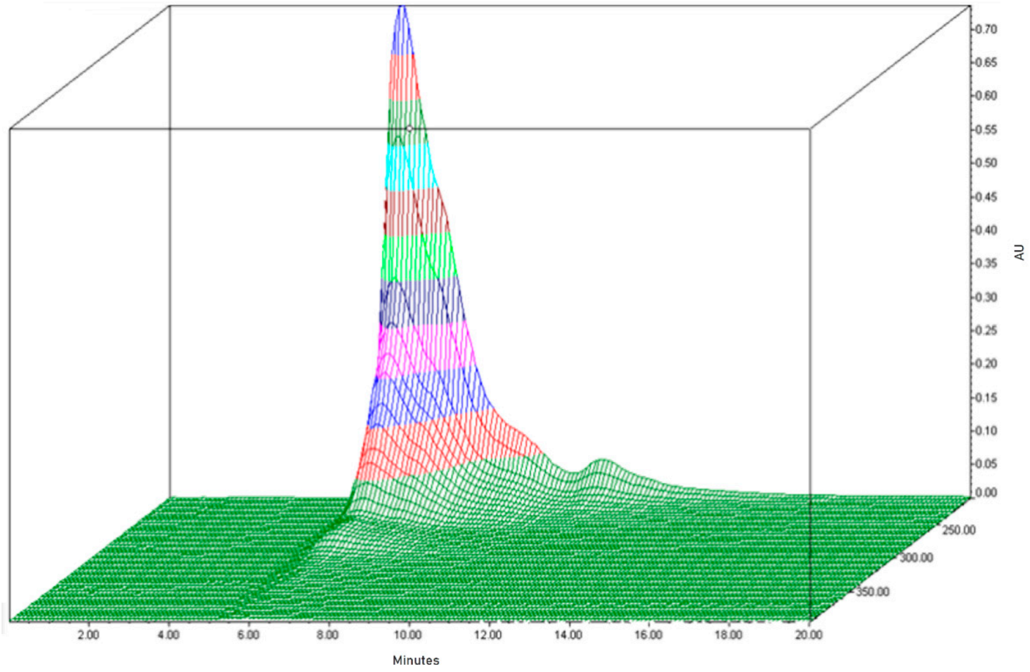


**Figure S1.** LDL chromatograms. The LDL-Dil complex bound to the fluorophore, which is seen in a retention time of 5.754 min. The fractions of native LDL extraction are shown and the fraction of the retention time of 5.75 minutes has been collected. All injections were made with a volume of 30 µl and under the same conditions using HPLC/UV/fluorescence kit (Waters, 2695/2996/2475).

**Table S1.** Differentially expressed genes. One hundred eighteen genes differentially expressed in men and women; Multiple Complex: 37, Coding: 37, Pseudogene: 2, Non-coding: 28, Precursor microRNA: 3, and Unassigned: 11.

| Transcript Cluster ID | Fold Change (linear) (MEN M1 vs. WOMEN M1) | ANOVA p-value (MEN M1 vs. WOMEN M1) | FDR p-value (MEN M1 vs. WOMEN M1) | Gene Symbol | Description | Group |
|---|---|---|---|---|---|---|
| TC0Y00007292.hg.1 | 50.37 | 0.00028 | 0.72114 | tybo | Transcript Identified by AceView | Coding |
| TC0Y00007309.hg.1 | 158.08 | 0.000508 | 0.810018 | blabo | Transcript Identified by AceView | Coding |
| TC0Y00006489.hg.1 | 81.27 | 0.000546 | 0.810018 | yohiru | Transcript Identified by AceView | Coding |
| TC0Y00007288.hg.1 | 64.22 | 0.000575 | 0.810018 | wubo | Transcript Identified by AceView | Coding |
| TC0Y00007296.hg.1 | 64.22 | 0.000575 | 0.810018 | sorbo | Transcript Identified by AceView | Coding |

| | | | | | | |
|---|---|---|---|---|---|---|
| TC0Y00007297.hg.1 | 64.22 | 0.000575 | 0.810018 | snubar | Transcript Identified by AceView | Coding |
| TC0Y00007301.hg.1 | 64.22 | 0.000575 | 0.810018 | shabo | Transcript Identified by AceView | Coding |
| TC0Y00007307.hg.1 | 64.22 | 0.000575 | 0.810018 | korbo | Transcript Identified by AceView | Coding |
| TC0Y00007305.hg.1 | 74.75 | 0.000713 | 0.810018 | merbo | Transcript Identified by AceView | Coding |
| TC0Y00007287.hg.1 | 50.55 | 0.000749 | 0.810018 | zobo | Transcript Identified by AceView | Coding |
| TC0Y00007299.hg.1 | 50.55 | 0.000749 | 0.810018 | skeybo | Transcript Identified by AceView | Coding |
| TC0Y00007303.hg.1 | 43.29 | 0.000789 | 0.810018 | sharbo | Transcript Identified by AceView | Coding |
| TC0Y00007308.hg.1 | 43.29 | 0.000789 | 0.810018 | blerbo | Transcript Identified by AceView | Coding |
| TC0Y00007290.hg.1 | 37.05 | 0.000865 | 0.810018 | vubo | Transcript Identified by AceView | Coding |
| TC0Y00007285.hg.1 | 38.49 | 0.000883 | 0.810018 | zeybu | Transcript Identified by AceView | Coding |
| TC0Y00007289.hg.1 | 57.68 | 0.000893 | 0.810018 | rarsybo | Transcript Identified by AceView | Coding |
| TC0Y00007294.hg.1 | 52.12 | 0.000939 | 0.810018 | tobo | Transcript Identified by AceView | Coding |
| TC0Y00007295.hg.1 | 71.5 | 0.000967 | 0.810018 | sybo | Transcript Identified by AceView | Coding |
| TC0Y00007300.hg.1 | 71.5 | 0.000967 | 0.810018 | shorbo | Transcript Identified by AceView | Coding |
| TC0Y00007302.hg.1 | 71.5 | 0.000967 | 0.810018 | pleybo | Transcript Identified by AceView | Coding |
| TC0Y00007304.hg.1 | 71.5 | 0.000967 | 0.810018 | pabo | Transcript Identified by AceView | Coding |
| TC0Y00007291.hg.1 | 28.29 | 0.001599 | 0.858212 | warbo | Transcript Identified by AceView | Coding |
| TC0Y00007298.hg.1 | 36.94 | 0.001631 | 0.85824 | skybor | Transcript Identified by AceView | Coding |
| TC0Y00007075.hg.1 | 4.18 | 0.001953 | 0.878309 | gyby | Transcript Identified by AceView | Coding |
| TC0Y00007076.hg.1 | 7.62 | 0.003481 | 0.878309 | lorby | Transcript Identified by AceView | Coding |
| TC0Y00007147.hg.1 | 8.25 | 0.005556 | 0.878309 | nabo | Transcript Identified by AceView | Coding |
| TC0900011076.hg.1 | 2.12 | 0.006732 | 0.878309 | plylo | Transcript Identified by AceView | Coding |
| TC1600009511.hg.1 | 2.21 | 0.00841 | 0.878309 | deysmoy | Transcript Identified by AceView | Coding |
| TC0400007713.hg.1 | -2.22 | 0.012279 | 0.878309 | STAP1 | signal transducing adaptor family member 1 | Coding |

| | | | | | | |
|---|---|---|---|---|---|---|
| TC1400006611.hg.1 | -2.01 | 0.016696 | 0.878309 | TRAV38-1 | T cell receptor alpha variable 38-1 | Coding |
| TC0600014101.hg.1 | 3.36 | 0.01675 | 0.878309 | MICA | MHC class I polypeptide-related sequence A | Coding |
| TC0500007703.hg.1 | 2.26 | 0.017098 | 0.878309 | peychaby | Transcript Identified by AceView | Coding |
| TC0500007711.hg.1 | 2.26 | 0.017098 | 0.878309 | cheychaby | Transcript Identified by AceView | Coding |
| TC0500012247.hg.1 | 3.17 | 0.022678 | 0.878309 | CD14 | CD14 molecule | Coding |
| TC1000008358.hg.1 | -3.02 | 0.030706 | 0.878309 | nihoro | Transcript Identified by AceView | Coding |
| TC0200008961.hg.1 | 3.16 | 0.034656 | 0.878309 | LOC654433 | Transcript Identified by AceView, Entrez Gene ID(s) 654433 | Coding |
| TC0Y00006919.hg.1 | 2.04 | 0.04827 | 0.878309 | serbar | Transcript Identified by AceView | Coding |
| TC0X00009007.hg.1 | -2.29 | 0.000196 | 0.72114 | PUDP | pseudouridine 5-phosphatase | Multiple_Complex |
| TC0Y00006632.hg.1 | 254.51 | 0.000206 | 0.72114 | DDX3Y | DEAD (Asp-Glu-Ala-Asp) box helicase 3, Y-linked | Multiple_Complex |
| TC0Y00006722.hg.1 | 2.92 | 0.000271 | 0.72114 | TXLNGY | taxilin gamma pseudogene, Y-linked | Multiple_Complex |
| TC0Y00006629.hg.1 | 4.73 | 0.000303 | 0.72114 | USP9Y | ubiquitin specific peptidase 9, Y-linked; testis-specific transcript, Y-linked 15 (non-protein coding) | Multiple_Complex |
| TC0Y00006730.hg.1 | 108.43 | 0.000384 | 0.778552 | EIF1AY | eukaryotic translation initiation factor 1A, Y-linked | Multiple_Complex |
| TC0Y00006487.hg.1 | 3.29 | 0.000465 | 0.805054 | ZFY | zinc finger protein, Y-linked | Multiple_Complex |
| TC0Y00006488.hg.1 | 8.3 | 0.000604 | 0.810018 | LINC00278 | long intergenic non-protein coding RNA 278 | Multiple_Complex |
| TC0400009086.hg.1 | -3.37 | 0.00074 | 0.810018 | GUCY1A3 | guanylate cyclase 1, soluble, alpha 3 | Multiple_Complex |
| TC0Y00007322.hg.1 | 44.91 | 0.001011 | 0.810018 | RPS4Y1 | ribosomal protein S4, Y-linked 1 | Multiple_Complex |
| TC0Y00007074.hg.1 | 23.23 | 0.001125 | 0.822304 | UTY | ubiquitously transcribed tetratricopeptide repeat containing, Y-linked | Multiple_Complex |
| TC0X00008945.hg.1 | -2.55 | 0.001133 | 0.822304 | PRKX | protein kinase, X-linked | Multiple_Complex |
| TC0X00011338.hg.1 | -2.66 | 0.001412 | 0.855519 | ARSD | arylsulfatase D | Multiple_Complex |

| | | | | | | |
|---|---|---|---|---|---|---|
| TC0Y00007160.hg.1 | 3.07 | 0.001745 | 0.86365 | KDM5D | lysine (K)-specific demethylase 5D | Multiple_Complex |
| TC0300013513.hg.1 | -3.01 | 0.001837 | 0.878309 | P3H2 | prolyl 3-hydroxylase 2 | Multiple_Complex |
| TC0500007114.hg.1 | -2.13 | 0.002698 | 0.878309 | RAI14 | retinoic acid induced 14 | Multiple_Complex |
| TC0Y00006644.hg.1 | 2.04 | 0.003397 | 0.878309 | ANOS2P | anosmin 2, pseudogene | Multiple_Complex |
| TC0Y00006651.hg.1 | 2.24 | 0.006646 | 0.878309 | NLGN4Y | neuroligin 4, Y-linked | Multiple_Complex |
| TC1500009377.hg.1 | -3.14 | 0.010176 | 0.878309 | ATP8B4 | ATPase, class I, type 8B, member 4 | Multiple_Complex |
| TC1200009585.hg.1 | -2.17 | 0.010805 | 0.878309 | RPS4XP14 | ribosomal protein S4X pseudogene 14 [Source:HGNC Symbol;Acc:HGNC:36737] | Multiple_Complex |
| TC0500011705.hg.1 | -2.46 | 0.010847 | 0.878309 | NREP | neuronal regeneration related protein | Multiple_Complex |
| TC0900008481.hg.1 | -2.1 | 0.01223 | 0.878309 | FAM225A | family with sequence similarity 225, member A (non-protein coding) | Multiple_Complex |
| TC14_GL000009v2_random00006455.hg.1 | -2.14 | 0.017308 | 0.878309 | RP11-435B5.4 | Salzman2013 ANNOTATED, ncRNA, OVERLAPTX, OVEXON best transcript TCONS_l2_00002643 | Multiple_Complex |
| TC1100007220.hg.1 | -2.17 | 0.021973 | 0.878309 | DEPDC7 | DEP domain containing 7 | Multiple_Complex |
| TC0800007065.hg.1 | -2.55 | 0.02362 | 0.878309 | CDCA2 | cell division cycle associated 2 | Multiple_Complex |
| TC1700007617.hg.1 | 3.33 | 0.025568 | 0.878309 | CCL18 | chemokine (C-C motif) ligand 18 | Multiple_Complex |
| TC14_GL000194v1_random00006432.hg.1 | -2.41 | 0.02639 | 0.878309 | MAFIP | MAFF interacting protein (pseudogene); tektin 4 pseudogene 2 | Multiple_Complex |
| TC0X00011259.hg.1 | -2.57 | 0.026458 | 0.878309 | STS | steroid sulfatase (microsomal), isozyme S | Multiple_Complex |
| TC1500007619.hg.1 | 2.41 | 0.027789 | 0.878309 | SMAD6 | SMAD family member 6 | Multiple_Complex |
| TC0400011015.hg.1 | 2.14 | 0.033086 | 0.878309 | CXCL3 | chemokine (C-X-C motif) ligand 3 | Multiple_Complex |
| TC1600011398.hg.1 | 10.03 | 0.033441 | 0.878309 | MMP2 | matrix metallopeptidase 2 | Multiple_Complex |
| TC0600006967.hg.1 | 2.85 | 0.033595 | 0.878309 | EDN1 | endothelin 1 | Multiple_Complex |
| TC0200008663.hg.1 | 2.41 | 0.03461 | 0.878309 | IL1R2 | interleukin 1 receptor, type II | Multiple_Complex |

| | | | | | | |
|---|---|---|---|---|---|---|
| TC1900010157.hg.1 | -2.73 | 0.03633 | 0.878309 | VN1R84P | vomeronasal 1 receptor 84 pseudogene | Multiple_Complex |
| TC0600011516.hg.1 | 3.52 | 0.043218 | 0.878309 | HLA-DOA | major histocompatibility complex, class II, DO alpha | Multiple_Complex |
| TC0400011018.hg.1 | 2.43 | 0.045651 | 0.878309 | CXCL2 | chemokine (C-X-C motif) ligand 2 | Multiple_Complex |
| TC0700006890.hg.1 | 5.94 | 0.046984 | 0.878309 | IL6 | interleukin 6 | Multiple_Complex |
| TC0100018434.hg.1 | -2.05 | 0.047389 | 0.878309 | MYCBP | MYC binding protein; gap junction protein alpha 9 | Multiple_Complex |
| TC0X00007813.hg.1 | 60.76 | 0.000105 | 0.72114 | RP13-348B13.2 | novel transcript | NonCoding |
| TC0X00010060.hg.1 | -1045.65 | 0.000232 | 0.72114 | XIST | X inactive specific transcript (non-protein coding) | NonCoding |
| TC0Y00006490.hg.1 | 150.81 | 0.000301 | 0.72114 | | | NonCoding |
| TC0Y00007073.hg.1 | 5.12 | 0.000304 | 0.72114 | | | NonCoding |
| TC0Y00007286.hg.1 | 55.36 | 0.000382 | 0.778552 | | | NonCoding |
| TC0Y00007306.hg.1 | 46.06 | 0.000425 | 0.778552 | | | NonCoding |
| TC0Y00007293.hg.1 | 91.68 | 0.000561 | 0.810018 | | | NonCoding |
| TC0Y00006529.hg.1 | 2.62 | 0.000696 | 0.810018 | Y_RNA | Y RNA [Source:RFAM;Acc:RF00019] | NonCoding |
| TC0Y00007159.hg.1 | 17.65 | 0.001175 | 0.833259 | RP11-424G14.1 | novel transcript | NonCoding |
| TC0300010593.hg.1 | -2.15 | 0.002982 | 0.878309 | | | NonCoding |
| TC1200010420.hg.1 | -2.28 | 0.003513 | 0.878309 | | | NonCoding |
| TC0Y00007072.hg.1 | 3.26 | 0.005109 | 0.878309 | | | NonCoding |
| TC2200007521.hg.1 | -5.95 | 0.00805 | 0.878309 | CTA-126B4.7 | novel transcript | NonCoding |
| TC0Y00006917.hg.1 | 4.61 | 0.008811 | 0.878309 | ZFY-AS1 | ZFY antisense RNA 1 [Source:HGNC Symbol;Acc:HGNC:38798]; novel transcript, antisense to ZFY | NonCoding |
| TC1200012378.hg.1 | 2.16 | 0.009409 | 0.878309 | | | NonCoding |
| TC1200006820.hg.1 | -2.04 | 0.014163 | 0.878309 | | | NonCoding |

| | | | | | | |
|---|---|---|---|---|---|---|
| TC0X00007617.hg.1 | -7.18 | 0.015015 | 0.878309 | TSIX | TSIX transcript, XIST antisense RNA | NonCoding |
| TC1000011186.hg.1 | 2.23 | 0.019222 | 0.878309 | | | NonCoding |
| TC14_GL000009v2_random00006446.hg.1 | -2.01 | 0.022008 | 0.878309 | | | NonCoding |
| TC0600014353.hg.1 | -2.51 | 0.024305 | 0.878309 | | | NonCoding |
| TC4_GL000008v2_random00006434.hg.1 | -2.65 | 0.026691 | 0.878309 | RP11-417J8.3 | | NonCoding |
| TC2100006511.hg.1 | -3.12 | 0.029916 | 0.878309 | | | NonCoding |
| TC2200006441.hg.1 | -3.12 | 0.029916 | 0.878309 | | | NonCoding |
| TC0X00008659.hg.1 | -2.02 | 0.036507 | 0.878309 | | | NonCoding |
| TC1900010095.hg.1 | -2.14 | 0.038497 | 0.878309 | | | NonCoding |
| TC1200012377.hg.1 | 3.07 | 0.038573 | 0.878309 | RP11-575F12.2 | novel transcript | NonCoding |
| TC0700009967.hg.1 | -2.07 | 0.040655 | 0.878309 | | | NonCoding |
| TC0600012920.hg.1 | 2.94 | 0.045604 | 0.878309 | LOC101927686 | uncharacterized LOC101927686; putative novel transcript; Transcript Identified by AceView | NonCoding |
| TC0600011419.hg.1 | -16.96 | 0.000587 | 0.810018 | MIR6891 | microRNA 6891 | Precursor_microRNA |
| TC2200006471.hg.1 | -2.25 | 0.008797 | 0.878309 | AC092854.1 | | Precursor_microRNA |
| TC4_GL000008v2_random00006437.hg.1 | -2.01 | 0.027639 | 0.878309 | AL583842.4 | | Precursor_microRNA |
| TC0Y00007169.hg.1 | 2.74 | 0.000284 | 0.72114 | RP11-256K9.1 | | Pseudogene |
| TC2000007828.hg.1 | -2.28 | 0.017022 | 0.878309 | RPS4XP3 | ribosomal protein S4X pseudogene 3 [Source:HGNC Symbol;Acc:HGNC:16251] | Pseudogene |
| TC0Y00006486.hg.1 | 9.9 | 0.000187 | 0.72114 | rorbor | Transcript Identified by AceView | Unassigned |
| TC0Y00006631.hg.1 | 3.28 | 0.001091 | 0.813972 | rawby | Transcript Identified by AceView | Unassigned |
| TC1700010167.hg.1 | 2.08 | 0.004789 | 0.878309 | pordy | Transcript Identified by AceView | Unassigned |

| | | | | | | |
|---|---|---|---|---|---|---|
| TC0X00009749.hg.1 | -3.39 | 0.006765 | 0.878309 | KDM5C | Transcript Identified by AceView, Entrez Gene ID(s) 8242 | Unassigned |
| TC1000011187.hg.1 | 2.12 | 0.015825 | 0.878309 | jeyshaw | Transcript Identified by AceView | Unassigned |
| TC1500006897.hg.1 | 3.23 | 0.02063 | 0.878309 | klawper | Transcript Identified by AceView | Unassigned |
| TC0Y00006630.hg.1 | 2.03 | 0.021083 | 0.878309 | nyby | Transcript Identified by AceView | Unassigned |
| TC0600009991.hg.1 | 2.17 | 0.032565 | 0.878309 | fyjaw | Transcript Identified by AceView | Unassigned |
| TC1600009555.hg.1 | 2.38 | 0.035234 | 0.878309 | sweeblo | Transcript Identified by AceView | Unassigned |
| TC0X00009137.hg.1 | -2.06 | 0.041192 | 0.878309 | buvar | Transcript Identified by AceView | Unassigned |
| TC0500011068.hg.1 | 2.23 | 0.044933 | 0.878309 | gusmarbu | Transcript Identified by AceView | Unassigned |

The Technical validation of the microarray with Q-PCR. In this panel we show some of the representative genes that had a difference in their expression in the different time intervals of LDL internalization. Q-PCR data correspond to the microarray and are shown adjusted for GAPDH expression illustratively.

**A)** 1. **B)** 2. **C)**

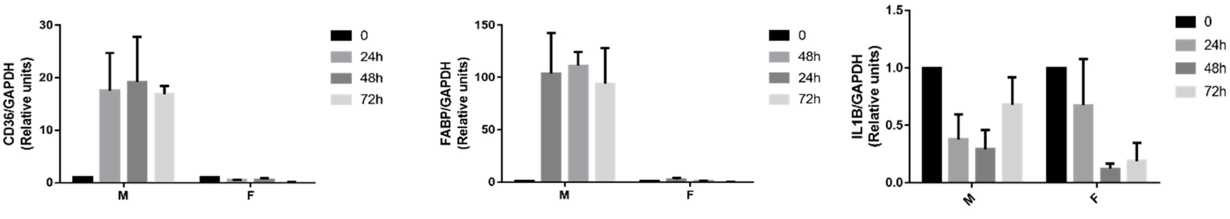


**Figure S2.** Technical validation of the microarray with Q-PCR. A) Expression of the CD36, function as a coreceptor for TLR4:TLR6 heterodimer, promotes inflammation in monocytes/macrophages. B) Expression of the FABP, plays a role in lipoprotein-mediated cholesterol uptake. C) Expression of the IL1B, this cytokine is produced by activated macrophages as a proprotein, which is proteolytically processed to its active form by caspase 1.
